# Subjective Sleep Quality in Relation to Relapse Status in Relapsing-Remitting Multiple Sclerosis: A Propensity Score—Matched Nested Case—Control Analysis

**DOI:** 10.3390/neurolint18100198

**Published:** 2026-10-21

**Authors:** Dimitar Taskov, Sonya Ivanova, Izabel Antova, Nikolay Topalov, Alexandra Barkalova-Atanasova, Nikolay Y. Yordanov, Ivan Milanov

**Affiliations:** 1 Multiprofile Hospital for Treatment in Neurology and Psychiatry St. Naum, 1113 Sofia, Bulgaria; 2 Department of Neurology, Medical University-Sofia, 1431 Sofia, Bulgaria

**Keywords:** multiple sclerosis, relapsing-remitting multiple sclerosis, sleep quality, Pittsburgh Sleep Quality Index, relapse, propensity score matching

## Abstract

**Background:** Sleep disturbance is common in multiple sclerosis (MS), but its relationship with relapse status remains insufficiently defined. This study examined whether poorer subjective sleep quality was associated with relapse status in patients with relapsing-remitting multiple sclerosis. **Methods:** This propensity score–matched nested case–control analysis included 132 patients with relapsing-remitting MS selected from 473 consecutively evaluated patients at a tertiary multiple sclerosis centre. Patients in relapse were matched 1:1 to patients in remission by age, sex, disease duration, Expanded Disability Status Scale score, disease-modifying therapy duration and efficacy class, and relapse in the previous year. Sleep quality and related symptoms were assessed using validated questionnaires. **Results:** Patients in relapse were more frequently classified as poor sleepers than those in remission (59.1% vs. 36.4%; *p* = 0.009). In the double-adjusted logistic regression model, higher Pittsburgh Sleep Quality Index scores were independently associated with relapse status (OR = 1.16; 95% CI 1.02–1.32; *p* = 0.023), whereas the remaining variables were not independently associated with relapse status. **Conclusions:** Poorer subjective sleep quality was independently associated with relapse status in relapsing-remitting MS, supporting sleep disturbance as a relapse-associated clinical feature rather than a proven causal risk factor.

## 1. Introduction

Multiple sclerosis (MS) is a chronic autoimmune disease of the central nervous system (CNS) characterised by inflammation, demyelination, axonal injury, and neurodegeneration. Relapsing–remitting multiple sclerosis (RRMS) is the most common form and is defined by clinical attacks (relapses) followed by periods of partial or complete recovery [1]. In a substantial proportion of patients, the disease eventually evolves into a progressive course. Despite significant advances in disease-modifying therapy (DMT), relapses continue to represent a substantial source of morbidity [1]. Understanding the factors associated with relapse activity remains a clinical priority.

Sleep is increasingly recognised as an important modulator of immune and neuroinflammatory processes [2]. Sleep alterations have been associated with dysregulation of cytokine signalling, glial homeostasis, and central nervous system clearance mechanisms. This may contribute to enhanced neuroinflammation and has been linked to disease activity in multiple sclerosis [3,4,5].

Sleep disturbance affects more than half of the patient population with MS but remains an underrecognised feature of this disease [6,7]. Poor sleep quality has been associated with greater disease burden, including higher relapse frequency, increased disability, and reduced quality of life [7,8]. Previous studies have focused mainly on sleep disturbance as a comorbidity or consequence of MS rather than a clinical feature associated with disease activity [9,10,11]. Preliminary evidence suggests that poor sleep quality may accompany periods of increased disease activity and is associated with higher relapse frequency and poorer long-term outcomes [12,13].

Whether poor sleep quality is associated with relapse status in patients with multiple sclerosis remains unclear. We therefore examined the associations between poor sleep quality, as measured by the Pittsburgh Sleep Quality Index (PSQI), and relapse status in patients with relapsing-remitting MS after controlling for key clinical and sleep-related factors.

## 2. Materials and Methods

### 2.1. Study Participants

This was a propensity score-matched nested case–control analysis within a single-centre cohort of patients with a confirmed diagnosis of MS who were consecutively evaluated at a tertiary multiple sclerosis centre in Sofia, Bulgaria, between February 2024 and March 2025. Diagnoses of MS were confirmed by board-certified neurologists according to the 2017 McDonald criteria [14]. The inclusion criteria were age ≥ 18 years, a confirmed diagnosis of RRMS, and current treatment with disease-modifying therapy. Patients with another form of MS, incomplete clinical data, other neurological or major psychiatric diseases or a prior diagnosis of sleep disorders were excluded from the analysis. The same eligibility criteria were applied to relapse and remission participants, except for relapse status at the time of assessment. Patients in relapse were evaluated within 72 h of symptom onset and before corticosteroid initiation. Relapse was defined as the occurrence of new or worsening neurological symptoms lasting at least 24 h, occurring in the absence of fever, infection, or other causes of pseudo-relapse, and separated from a previous relapse by at least 30 days. Relapses were confirmed clinically by board-certified neurologists. MRI was performed when clinically indicated but was not required for relapse confirmation. The participants in the control group were selected from a previously published cohort of 399 patients with RRMS in remission evaluated at the same study centre [15]. The remission cohort was assessed using the same clinical approach and the same sleep- and symptom-related instruments as the relapse group.

### 2.2. Clinical Evaluation

A neurologist with experience in demyelinating diseases evaluated all patients. Demographic and clinical variables, including age, sex, and neurological disability assessed by the Expanded Disability Status Scale (EDSS), were recorded [16]. For patients in the relapse group, the EDSS score was determined on the basis of their last documented stable neurological examination before the current exacerbation. Additional data included disease duration, history of relapse in the previous year, and DMT characteristics. This included duration of current treatment and its efficacy class: moderate (interferons, glatiramer acetate, teriflunomide or dimethyl fumarate) or high (fingolimod, natalizumab, alemtuzumab, ocrelizumab, ofatumumab or cladribine).

### 2.3. Evaluation of Sleep Quality and Related Symptoms

Subjective sleep quality and related symptoms were assessed using validated self-report instruments. The Pittsburgh Sleep Quality Index (PSQI) is used to evaluate sleep quality during the previous month, with scores > 5 indicating poor sleep [17]. The Insomnia Severity Index (ISI) was used to assess insomnia symptoms over the past two weeks [18]. The Fatigue Severity Scale (FSS) reflects fatigue over the past few weeks [19]. The Epworth Sleepiness Scale (ESS) was used to assess the likelihood of falling asleep during normal daily activities [20]. The STOP-Bang questionnaire was used to assess the risk of obstructive sleep apnea (OSA) [21]. The Hamilton Anxiety Rating Scale (HAM-A) was used to measure current anxiety severity [22]. Higher scores on all scales indicated greater symptom burden.

All questionnaires were administered in Bulgarian. The Bulgarian versions were obtained from their respective copyright holders or the MAPI Research Trust. The same questionnaire versions were used in both relapse and remission groups. The use of these instruments and their psychometric properties in Bulgarian patients with RRMS has been previously reported [15].

Although the PSQI was administered according to its standard one-month reference period, this interval may have overlapped with prodromal or early relapse-related symptoms in patients evaluated during relapse. Therefore, overlap between poor sleep quality and the early clinical or prodromal phase of relapse cannot be excluded. This limits causal and temporal interpretation and may have introduced recall or reverse-causation bias.

### 2.4. Statistical Analysis

The data were analysed via IBM SPSS Statistics version 23.0 (IBM Corp., Armonk, NY, USA). Statistical significance was defined as two-tailed *p* < 0.05. Continuous variables (age, EDSS score, disease duration, DMT duration, and questionnaire scores) were tested for normality using the Shapiro–Wilk test. Normally distributed variables are presented as means ± standard deviations (SDs), and nonnormally distributed variables are presented as medians and interquartile ranges (IQRs). Categorical variables are presented as counts and percentages. Between-group comparisons (relapse vs. remission) were performed using the independent-samples t-test or the Mann–Whitney U test for continuous variables, and the chi-square test or Fisher’s exact test for categorical variables, as appropriate. Effect sizes were calculated for the main between-group comparisons. For non-normally distributed continuous variables, rank-biserial correlation was used, while odds ratios were used for categorical outcomes.

Propensity score matching (PSM) was applied to identify appropriate remission controls from a previously published cohort of 399 patients with RRMS in remission [15]. These participants were matched to MS patients in relapse based on key demographic and clinical characteristics to minimise confounding and achieve well-balanced comparison groups. Propensity scores were estimated using binary logistic regression including age, sex, EDSS score, disease duration, DMT duration, DMT efficacy class, and the presence of relapse in the previous year. Model discrimination and calibration were evaluated prior to matching using the C statistic and the Hosmer–Lemeshow goodness-of-fit test, respectively. Adequate model performance was defined as a C-statistic ≥ 0.7 and a Hosmer–Lemeshow *p* > 0.05. A 1:1 nearest-neighbour matching without replacement, with a calliper < 0.1, was used. Covariate balance was evaluated using standardised mean differences (SMDs), with values less than 0.10 considered optimal and values less than 0.20 considered acceptable. To assess potential selection bias introduced by matching, relapse patients included in the matched analysis were compared with relapse patients who could not be matched. The design, implementation, and reporting of PSM followed established methodological recommendations for multiple sclerosis research [23,24] and general epidemiological standards for post–matching adjustment [25]. After matching, a double–adjusted logistic regression model was used to estimate the independent association between sleep quality (PSQI score) and relapse status. The model additionally controlled for fatigue and anxiety, as clinically relevant symptom domains related to sleep quality and relapse-related burden. Covariates with residual imbalance after matching (SMD > 0.10) were also included. Multicollinearity was assessed using variance inflation factors (VIFs), with values less than 5 considered acceptable for inclusion. Because propensity score matching generated 66 matched relapse–remission pairs, a sensitivity analysis was performed using conditional logistic regression with a matched pair as the stratification variable. This analysis was used to assess whether the association between PSQI score and relapse status remained consistent after explicitly accounting for the matched design.

## 3. Results

### 3.1. Participants

We screened 473 RRMS patients—74 during relapse and 399 in remission. After PSM, eight patients who experienced relapse could not be matched and were excluded from the analyses. Finally, 132 patients (66 relapse–remission pairs) were included in the analysis.

Potential selection bias introduced by matching was assessed by descriptively comparing relapse patients included in the matched analysis with those who could not be matched. Matched and unmatched relapse patients were generally comparable in demographic and clinical characteristics. However, unmatched relapse patients exhibited higher PSQI, ISI, and FSS scores than matched relapse patients (Supplementary Table S1).

The propensity score model demonstrated good discrimination (C-statistic = 0.734) and acceptable calibration (Hosmer–Lemeshow *p* = 0.21). Post-matching covariate balance was substantially improved. All covariates achieved optimal balance (SMD < 0.10), except for age, which showed only minimal residual imbalance (SMD = 0.11) (Table 1).

### 3.2. Group Comparison

After matching, no significant differences were observed in demographic or clinical parameters, including age, sex, disease duration, EDSS score, DMT duration, or DMT efficacy class (all *p* > 0.05) (Table 2). In contrast, several patient-reported outcomes differed significantly between the groups. Patients in the relapse group were more frequently categorised as poor sleepers than patients in remission (59.1% vs. 36.4%; *p* = 0.009) and had higher median PSQI scores (6 vs. 4, *p* = 0.001). The odds of being classified as a poor sleeper were higher in the relapse group (OR = 2.53; 95% CI 1.25–5.10). The between-group difference in PSQI score corresponded to a small-to-moderate effect size (rank-biserial correlation = 0.29). Additionally, patients who experienced relapse reported greater fatigue severity (FSS, *p* = 0.047) and higher anxiety levels (HAM-A, *p* = 0.030). No statistically significant differences in insomnia severity (ISI), daytime sleepiness (ESS), or OSA risk (STOP-Bang) were found between the two groups (Table 2).

### 3.3. Double–Adjusted Regression Analysis

The double–adjusted logistic regression model indicated that higher PSQI scores were independently associated with relapse status (OR = 1.16; 95% CI 1.02–1.32; *p* = 0.023). None of the other covariates, including age, FSS score, and HAM-A scores, were significantly associated with relapse status. The model demonstrated acceptable goodness-of-fit (Hosmer–Lemeshow *p* = 0.124) but limited explanatory power (R^2^ = 0.095). Multicollinearity testing demonstrated no significant overlap between predictors, as all VIF values were <2 (Table 3).

Sensitivity analysis using conditional logistic regression stratified by matched pair showed that higher PSQI scores remained associated with relapse status (OR = 1.16; 95% CI 1.02–1.33; *p* = 0.027). FSS score, HAM-A score, and age were not significantly associated with relapse status in this model.

## 4. Discussion

In this propensity score-matched nested case–control analysis, poorer subjective sleep quality was independently associated with relapse status (OR = 1.16 per point; 95% CI 1.02–1.32; *p* = 0.023) after controlling for key clinical and sleep-related measures. This association remained consistent in a sensitivity analysis using conditional logistic regression that accounted for the matched-pair design. In contrast, insomnia severity (ISI), daytime sleepiness (ESS), and OSA risk (STOP-Bang) did not differ significantly between the groups. These findings suggest that poorer subjective sleep quality is part of the relapse-associated clinical profile in RRMS. However, the present design does not allow determination of whether poor sleep precedes relapse, reflects a prodromal phase, or occurs as part of the early relapse-related symptom burden.

Our results are consistent with previous studies reporting an association between sleep disturbances and relapse-related outcomes in patients with MS. For example, Buratti et al. reported that poor sleep quality (PSQI > 5) was associated with a greater number and duration of relapses [12]. Furthermore, Sahraian et al. reported that sleep disorders frequently accompany acute MS exacerbations [13]. However, available evidence on the relationship between sleep quality and MS disease activity remains mixed. In a recent multidisciplinary cross-sectional study, no significant differences were found between patients with good and poor sleep quality in terms of average annual relapse rate, EDSS score, disease duration, DMT duration, or DMT type [26]. Similarly, a recent PSQI-based meta-analysis reported substantial heterogeneity across studies and did not find a statistically significant difference in the prevalence of poor sleep quality between patients with MS and healthy controls, despite higher PSQI scores in patients with MS [11]. These discrepancies suggest that the relationship between sleep quality and MS disease activity is complex and may be influenced by overall symptom burden, including fatigue, anxiety and depressive symptoms, pain, disability, and reduced quality of life [27,28]. Our study extends these findings by focusing specifically on current relapse status and by applying propensity score matching to minimise confounding by demographic and clinical factors, including age, sex, disease duration, disability, DMT efficacy class, DMT duration, and prior relapse.

Several biological mechanisms may provide plausibility for the observed association between sleep disturbance and relapse status in MS, including immune dysregulation and cellular reparative mechanisms. Sleep disruption has been associated with elevations in proinflammatory cytokines such as interleukin-6 (IL-6) and tumour necrosis factor α (TNF-α). It may also compromise blood–brain barrier integrity and activate glial cells [12,13]. Adequate sleep supports immune homeostasis, glial stability, and oligodendrocyte precursor proliferation—processes essential for remyelination and recovery [29]. Impairment of the glymphatic system, a sleep-dependent clearance mechanism, can lead to the accumulation of inflammatory metabolites and astrocytic activation, further sustaining neuroinflammation and neurodegeneration [3,30]. Conversely, relapse-related symptoms such as pain, nocturia, spasticity, reduced mobility, and anxiety may themselves worsen perceived sleep quality during the prodromal or early relapse period. These mechanisms support a potentially bidirectional relationship between poor sleep quality and relapse-related symptom burden, without establishing causality.

The observed association supports consideration of sleep quality as part of the broader symptom assessment of patients with MS during relapse evaluation and follow-up. The use of validated subjective sleep-related instruments, such as the PSQI, may help characterise symptom burden and relapse-associated clinical profiles in routine practice. Although behavioural interventions targeting sleep quality, including cognitive behavioural therapy for insomnia, sleep hygiene, and physical exercise, have been shown to improve sleep quality, fatigue, and mood, their potential impact on relapse-related outcomes requires confirmation in prospective studies [31].

One of the strengths of this study is the use of propensity score matching to control for key demographic and clinical confounders, resulting in well-balanced study cohorts. The use of easy-to-use and validated instruments to assess sleep quality and related symptoms further increases the reliability of the findings. However, several limitations should be considered. First, the nested case–control design precludes causal inference. Although the PSQI was administered using its standard one-month reference period, this period may have overlapped with prodromal or early relapse-related symptoms in patients assessed during relapse. Therefore, the possibility of overlap between poor sleep quality and the early clinical or prodromal phase of relapse cannot be excluded. This limits causal and temporal interpretation and may have introduced recall or reverse-causation bias. Second, although both matched and unmatched relapse patients were compared to assess possible selection bias, the unmatched group was small. Unmatched relapse patients demonstrated higher PSQI, ISI, and FSS scores than matched relapse patients, suggesting that excluding these cases may have led to a conservative estimate of the association between relapse status and subjective sleep quality. However, because only eight relapse patients remained unmatched, this observation should be interpreted with caution. Third, sleep quality was evaluated using subjective questionnaires, whereas objective measures such as actigraphy and polysomnography may provide more robust evidence. Fourth, depression and pain were not assessed with dedicated instruments. Residual confounding from other unmeasured factors, including medication effects, lifestyle factors, undiagnosed sleep-related comorbidities, and relapse-related symptoms such as bladder dysfunction or spasticity cannot be excluded. Finally, although relapse topography and predominant neurological symptoms were documented clinically, they were not analysed in relation to sleep outcomes, preventing evaluation of whether sleep disturbance varied according to relapse type.

## 5. Conclusions

In conclusion, our findings show that poorer subjective sleep quality was independently associated with relapse status in patients with RRMS. This finding suggests that sleep disturbance is a relapse-associated clinical feature rather than a proven causal risk factor. Future prospective longitudinal studies that include objective sleep measures are needed to clarify the temporal relationship between sleep disturbance and relapse status in patients with RRMS.

## Figures and Tables

**Table 1:** Covariate balance before and after propensity score matching.

Covariate	SMD Before Matching *N* = 473	SMD After Matching *N* = 132
Age	0.07	0.11
Sex	0.05	0.03
Disease duration	0.34	0.05
EDSS	0.38	0.07
Relapse in the past year	0.04	0.03
DMT duration	0.11	0.09
High-efficacy DMT	0.14	0.07

Abbreviations: SMD—Standardised Mean Difference; EDSS—Expanded Disability Status Scale; DMT—Disease-modifying therapy.

**Table 2:** Clinical and patient-reported characteristics. Continuous data are presented as mean ± standard deviation (SD) or median (interquartile range, IQR), depending on distribution. Categorical data are shown as counts and percentages. *p*-values were obtained using *t*-test, Mann–Whitney U, or χ^2^ test, as appropriate.

Variable	Relapse (*n* = 66)	Remission (*n* = 66)	*p*-Value
Age	40.6 ± 9.9	39.4 ± 9.5	0.49
Female sex, *n* (%)	47 (71.2)	45 (68.2)	0.71
Disease duration, years	9.4 (4–13)	9.7 (5–12)	0.79
EDSS	3.2 (2.5–4.0)	3.1 (2.0–4.0)	0.69
Relapse in the last year	10 (15.2)	11 (16.7)	0.81
DMT duration, years	7.4 (3–10)	6.9 (3–9)	0.59
High–efficacy DMT, *n* (%)	13 (19.7)	15 (22.7)	0.67
PSQI score	6 (4–8)	4 (2–6)	**0.001**
Poor sleeper (PSQI > 5), *n* (%)	39 (59.1)	24 (36.4)	**0.009**
Insomnia Severity Index	7 (4–10)	6 (3–9)	0.13
Fatigue Severity Scale	33.5 (25–46)	29 (22–35)	**0.047**
Epworth Sleepiness Scale	6 (3–8)	5 (3–7)	0.08
Hamilton Anxiety Rating Scale	14 (8–21)	12 (7–18)	**0.03**
STOP-Bang	1 (1–2)	1 (0–2)	0.21

Abbreviations: EDSS—Expanded Disability Status Scale; DMT—Disease-modifying therapy; PSQI—Pittsburgh Sleep Quality Index. Continuous data are presented as mean ± standard deviation (SD) or median (interquartile range, IQR), depending on distribution. Categorical data are shown as counts and percentages. *p*-values were obtained using *t*-test, Mann–Whitney U test, or χ^2^ test, as appropriate. Bold font indicates statistically significant results (*p* ≤ 0.05).

**Table 3:** Double–adjusted logistic regression model for associations with relapse status.

Predictor	OR (95% CI)	B	SE	Wald	*p*-Value
PSQI score	1.16 (1.02–1.32)	0.148	0.065	5.21	**0.023**
FSS	1.00 (0.97–1.03)	0.003	0.015	0.05	0.822
HAM-A	1.01 (0.96–1.06)	0.006	0.024	0.06	0.812
Age	1.00 (0.96–1.04)	−0.001	0.019	0.00	0.968

Abbreviations: OR—odds ratio; SE—standard error; CI—confidence interval; PSQI—Pittsburgh Sleep Quality Index; FSS—Fatigue Severity Scale; HAM-A—Hamilton Anxiety Rating Scale. Odds ratios (OR) are expressed per one-point increase in questionnaire scores. Bold font indicates statistically significant results (*p* ≤ 0.05).

## Data Availability

The data presented in this study are available on request from the corresponding author.

## Supplementary Materials

The following supporting information can be downloaded at: https://www.mdpi.com/article/10.3390/neurolint18100198/s1, Table S1: Descriptive comparison of matched and unmatched relapse-patients.

## Abbreviations

The following abbreviations are used in this manuscript:

CI	Confidence interval
CNS	Central nervous system
DMT	Disease-modifying therapy
EDSS	Expanded Disability Status Scale
ESS	Epworth Sleepiness Scale
FSS	Fatigue Severity Scale
HAM-A	Hamilton Anxiety Rating Scale
IL-6	Interleukin-6
ISI	Insomnia Severity Index
IQR	Interquartile range
MS	Multiple sclerosis
OR	odds ratio
PSQI	Pittsburgh Sleep Quality Index
PSM	Propensity score matching
RRMS	Relapsing-remitting multiple sclerosis
SDs	Standard deviations
SMDs	Standardised mean differences
TNFα	Tumour necrosis factor α
VIFs	Variance inflation factors
