# Supplementary material for: Subjective Sleep Quality in Relation to Relapse Status in Relapsing-Remitting Multiple Sclerosis: A Propensity Score—Matched Nested Case—Control Analysis
Source: Neurol Int. 2026 Oct 21;18(10):198. doi: 10.3390/neurolint18100198 (PMC13708224; doi:10.3390/neurolint18100198)
Supplement: Supplementary file 1 [file neurolint-18-00198-s001.zip › neurolint-4593579-supplementary.pdf]

Table S1 Descriptive comparison of matched and unmatched relapse-patients.

Variable	Matched relapse patients (n=66)	Unmatched relapse patients (n=8)	p-value
Age	40.61 ± 9.94	44.00 ± 12.47	0.38
Female sex, n (%)	47 (71.2)	7 (87.5)	0.43
Disease duration, years	7.5 (4.0–13.8)	4.0 (1.8–11.0)	0.45
EDSS	3.0 (2.5–4.0)	5.5 (2.8–6.1)	0.07
DMT duration, years	7.44 (3–10)	6.91 (3–9)	0.87
Relapse in previous year, n (%)	10 (15.2)	2 (25.0)	0.61
High-efficacy DMT, n (%)	13 (19.7)	2 (25)	0.67
PSQI score	6.0 (4–9)	10.5 (7–12)	**0.045**
Insomnia Severity Index	6 (2.0–10.0)	13.5 (9–15)	**0.009**
Fatigue Severity Scale	31 (24–45)	55 (44–60)	**0.001**
Epworth Sleepiness Scale	6 (3–8)	8 (6–10)	0.09
Hamilton Anxiety Rating Scale	14 (9–20)	19 (13–24)	0.15
STOP-Bang	1 (1–2)	2 (1–2)	0.44

Abbreviations: EDSS—Expanded Disability Status Scale; DMT—Disease-modifying therapy; PSQI—Pittsburgh Sleep Quality Index. Continuous data are presented as mean ± standard deviation or median (interquartile range), depending on distribution. Categorical data are shown as counts and percentages. P-values were obtained using the t–test, Mann–Whitney U test, or χ^2 test, as appropriate. Bold font indicates statistically significant results ($p \leq 0.05$).
